# Tranexamic acid use decreases transfusion rate in children with cerebral palsy undergoing proximal femoral varus derotational osteotomy

**DOI:** 10.1097/MD.0000000000028506

**Published:** 2022-01-14

**Authors:** Edward Compton, Rachel Y. Goldstein, Alexander Nazareth, Stephen J. Shymon, Lydia Andras, Robert M. Kay

**Affiliations:** a Children's Orthopaedic Center, Children's Hospital Los Angeles, Los Angeles, CA; b Department of Orthopaedic Surgery, Harbor-UCLA Medical Center, Torrance, CA; c Department of Anesthesiology, Children's Hospital Los Angeles, Los Angeles CA.

**Keywords:** cerebral palsy, proximal femoral varus derotational osteotomy, tranexamic acid

## Abstract

Previous studies demonstrated the safety of tranexamic acid (TXA) use in cerebral palsy (CP) patients undergoing proximal femoral varus derotational osteotomy (VDRO), but were underpowered to determine if TXA alters transfusion rates or estimated blood loss (EBL). The purpose of this study was to investigate if intraoperative TXA administration alters transfusion rates or EBL in patients with CP undergoing VDRO surgery.

We conducted a retrospective review of 390 patients with CP who underwent VDRO surgery between January 2004 and August 2019 at a single institution. Patients without sufficient clinical data and patients with preexisting bleeding or coagulation disorders were excluded. Patients were divided into 2 groups: those who received intraoperative TXA and those who did not.

Out of 390 patients (mean age 9.4 ± 3.8 years), 80 received intravenous TXA (TXA group) and 310 did not (No-TXA group). There was no difference in mean weight at surgery (*P* = .25), Gross Motor Function Classification System level (*P* = .99), American Society of Anesthesiologist classification (*P* = .50), preoperative feeding status (*P* = .16), operative time (*P* = .91), or number of procedures performed (*P* = .12) between the groups. The overall transfusion rate was lower in the TXA group (13.8%; 11/80) than the No-TXA group (25.2%; 78/310) (*P* = .04), as was the postoperative transfusion rate (7.5%; 6/80 in the TXA group vs 18.4%; 57/310 in the No-TXA group) (*P* = .02). The intraoperative transfusion rate was similar for the 2 groups (TXA: 7.5%; 6/80 vs No-TXA: 10.3%; 32/310; *P* = .53). The EBL was slightly lower in the TXA group, although this was not significant (TXA: 142.9 ± 113.1 mL vs No-TXA: 177.4 ± 169.1 mL; *P* = .09). The standard deviation for EBL was greater in the No-TXA group due to more high EBL outliers. The percentage of blood loss based on weight was similar between the groups (TXA: 9.2% vs No-TXA: 10.1%; *P* = .40). The number needed to treat (NNT) with TXA to avoid one peri-operative blood transfusion in this series was 9.

The use of intraoperative TXA in patients with CP undergoing VDRO surgery lowers overall and postoperative transfusion rates.

Level of evidence: III, Retrospective Comparative Study.

## 1 Introduction

Cerebral palsy (CP) is a nonprogressive insult to the brain during development resulting in motor deficits and activity limitation.^[1,2]^ CP is the most common cause of persistent motor deficit in children, with an estimated prevalence of 2 to 3.3 per 1000 live births.^[3,4]^ Upwards of 85% of children with CP experience muscle spasticity, often with strong contraction of the hip adductors, which results in abnormal proximal femoral anatomy and hip subluxation or dislocation in approximately 30% to 35% of children with CP.^[3–5]^ Bony and soft-tissue procedures such as pelvic osteotomies, hip reconstructions, and hamstring lengthening are often indicated to restore normal anatomy.^[5–7]^

Patients with CP have a propensity for increased blood loss during surgery due to seizure medications, poor nutritional status, abnormal platelet function and connective tissue properties, and depletion of clotting factors.^[8–11]^ Patients undergoing hip reconstructions can sustain substantial blood loss, with previous studies estimating blood loss (EBL) between 150 and 970 mL.^[12–14]^ Subsequently, in patients with CP undergoing hip reconstructive surgery, transfusion rates have been reported between 19% and 67%.^[12,14,15]^ Efforts to minimize allogenic blood transfusions have been made, as these transfusions carry the potential risk of bacterial or viral infections, hemolytic reactions, transfusion related acute lung injury, and death.^[16–18]^ Tranexamic acid (TXA) is a synthetic lysine analogue that acts as an antifibrinolytic agent by binding to lysine binding sites on plasminogen. This prevents plasminogen from interacting with lysine residues on fibrin, resulting in inhibition of clot dissolution and hemostasis.^[19,20]^ TXA has successfully been used in total joint arthroplasty and spine surgery to minimize blood loss and reduce allogenic blood transfusion.^[19,21–26]^

The literature on TXA use in patients with CP undergoing orthopaedic procedures is limited, with few studies documenting the safety and efficacy of TXA in this patient population. The purpose of this study was to investigate the efficacy of intraoperative intravenous TXA administration in reducing blood loss and transfusion requirements in patients with CP undergoing proximal femoral varus derotational osteotomy (VDRO) surgery. We sought to characterize intraoperative and postoperative transfusion rates, intra-operative blood loss, postoperative hemoglobin and hematocrit levels, and length of inpatient stay between patients with CP treated with and without TXA during VDRO surgery. We hypothesized that patients receiving TXA would have lower intraoperative blood loss and a lower transfusion requirement than patients not receiving TXA.

## 2 Methods

Institutional review board approval was granted for this study. All patients with CP who underwent VDRO surgery at the authors’ institution, a tertiary referral center, between January 2, 2004 and August 22, 2019 were retrospectively identified. The first use of TXA in patients with CP undergoing VDRO at our institution was January 8, 2010, but more routine TXA use during VDRO surgery began at our institution in 2014 after its reported efficacy in the literature. From 2014 until the conclusion of the study period, approximately half of such patients received TXA (63/130). TXA administration was at the discretion of the treating surgeon and anesthesiologist. Patients were included if they underwent a VDRO for CP during the study period. Patients without sufficient clinical data and patients with preexisting bleeding or coagulation disorders were excluded.

Patients underwent VDRO with additional bony and/or soft tissue procedures in the same surgical session as indicated.^[27]^ Patients were divided into 2 groups: those who received intra-operative TXA (TXA group; n = 80) and those who did not (No-TXA group; n = 310). TXA was administered at a loading dose followed by a maintenance dose. The dosing of TXA in our institution has decreased over time. Initially, when we began using TXA, the patients typically received a loading dose of 50 mg/kg, following by a maintenance dose of 5 to 10 mg/kg/hour throughout surgery. Currently, a typical loading dose is 15 to 20 mg/kg, followed by maintenance of 5 mg/kg/hour for the remainder of surgery. The majority of patients underwent a combination of general and spinal anesthesia. Postoperative drains were not routinely placed.

Demographic data including sex, age, height, weight, comorbid conditions, Gross Motor Function Classification System (GMFCS), American Society of Anesthesiologists physical status (ASA), preoperative feeding status, and preoperative medication use were collected. Patients at GMFCS levels I, II, and III were considered ambulatory, while patients at GMFCS levels IV and V were considered non-ambulatory. Surgical data included number and type of additional procedures, length of surgery and anesthesia, fluid administration, blood product transfusion, urine output, and tourniquet time.^[28,29]^ At the conclusion of surgery, blood loss was estimated via surgeon and anesthesiologist agreement based on inspection of suction and surgical sponges and recorded in the anesthesia records. Blood loss was also estimated using the hemoglobin balance method,^[30–33]^ where:

EBL = 1000 × (Hb_loss__total_)/Hb_i_
Hb_loss__total_= BV × (Hb_i_-Hb_f_) x 0.001 + Hb_t_
BV = Blood volume(mL) = 70 mL/kg × Weight (kg); Hb_i_ (g/L) = preoperative Hb value; Hb_f_ (g/L) = postoperative Hb value; Hb_t_ (g) = the amount of Hb transfused; 1 unit of banked blood was assumed to have 52 g of Hb^[32]^

Preoperative hemoglobin and hematocrits were measured within 30 days of the procedure. At our institution, there is no standard protocol requiring postoperative laboratory monitoring. Patients with low EBL relative to their total blood volume (TBV), and good clinical and hemodynamic stability do not always undergo blood draws postoperatively, and therefore, not all patients had a postoperative hematocrit and hemoglobin measured. Intraoperative and postoperative transfusions were recorded. Transfusions were typically initiated after serial labs demonstrated down-trending hematocrit and/or hemoglobin levels (typically with thresholds for transfusion of a hemoglobin <7 g/dL and/or a hematocrit <20%), or if a patient had symptoms of anemia (orthostatic hypotension, tachycardia unresponsive to fluid resuscitation, dizziness, etc.). Final follow-up was defined as the most recent clinical visit.

Continuous outcome variables were analyzed using unpaired Student *t* tests and categorical variables were analyzed using Fisher exact test. A multiple logistic regression was performed to determine the association of TXA use and transfusion rate, while controlling for unilateral vs bilateral VDROs, pelvic osteotomies, and ambulatory status. Relative risk (RR) of transfusion was calculated with 95% confidence intervals (CI). The number needed to treat was calculated as 1/Absolute Risk Reduction. Statistical significance was defined as *P* < .05. Statistical analysis was performed using STATA/1C 14.0 (Stata Statistical Software:Release 14; StataCorp LP, 2015, College Station, TX).

## 3 Results

Three hundred ninety patients met inclusion criteria. Of these, 80 were in the TXA group and 310 were in the No-TXA group. The average dose of TXA in the TXA group was 54.0 ± 32.3 mg/kg. Weight at surgery (*P* = .25), GMFCS level (*P* = .99), ASA physical status (*P* = .50), preoperative feeding status (*P* = .16), number of concomitant procedures performed (*P* = .12), and prevalence of concomitant pelvic osteotomies (*P* = .32) were similar between the 2 groups (Table 1). There were significantly more males in the No-TXA group (64.5%; 200/310) than in the TXA group (47.5%; 38/80) (*P* = .007; Table 1). Patients in the No-TXA group (120.0 ± 24.0 cm) were significantly taller than patients in the TXA group (113.8 ± 24.1 cm) (*P* = .046; Table 1). Patients in the TXA group (62.5%; 50/80) were more likely to use preoperative seizure medications than patients in the No-TXA group (41.0%; 127/310) (*P* = .001; Table 1). Patients in the TXA group (83.8%; 67/80) were more likely to undergo a bilateral VDRO than patients in the No-TXA group (68.7%; 213/310) (*P* = .008; Table 1). Baseline hemoglobin and hematocrit values were not different between the 2 groups (*P* = .27 and *P* = .61, respectively; Table 2).

**Table 1:** Baseline information of cerebral palsy patients undergoing proximal femoral varus derotational osteotomies treated with and without tranexamic acid.

Characteristic	No-TXA (n = 310)	TXA (n = 80)	*P* value
Gender, n (%)			
Male	200 (65)	38 (48)	
Female	110 (35)	42 (52)	.007^∗^
Weight at surgery, kg	26.6 (14.0)	24.6 (13.3)	.25
Height at surgery, cm	120.0 (24.0)	113.8 (24.1)	.046^∗^
GMFCS	I: 6	I: 1	
	II: 33	II: 9	
	III: 40	III: 10	.99
	IV: 125	IV: 33	
	V: 106	V: 27	
ASA Physical status	I: 4	I: 0	
	II: 90	II: 21	
	III: 215	III: 58	.50
	IV: 1	IV: 1	
Preoperative feeding status	Oral: 230	Oral: 51	
	G-tube: 69	G-tube: 26	
	GJ-tube: 4	GJ-tube: 2	.16
	Combined Oral/G-tube: 7	Combined Oral/G-tube: 1	
Preoperative seizure medication use	Yes: 127	Yes: 50	
	No: 183	No: 30	.001^∗^
VDRO side	Bilateral: 213	Bilateral: 67	.008^∗^
	Unilateral: 97	Unilateral: 13	
Pelvic osteotomy	Yes: 56	Yes: 10	
	No: 254	No: 70	.32
Procedures performed	6.1 (2.8)	5.6 (2.2)	.12
Operative time, minutes	232.8 (117.9)	234.5 (121.4)	.91

Values are presented as mean (standard deviation) unless otherwise noted; *P* values were determined using unpaired *t* tests and Fisher exact test for continuous and categorical variables, respectively.

∗ indicates significance at the *P* < .05 level. ASA = American Society of Anesthesiologists; G-tube = gastrostomy tube; GJ-tube = gastro-jejunal tube; GMFCS = Gross Motor Function Classification System; VDRO = varus derotational osteotomy

**Table 2:** Pre- and postoperative laboratory values, transfusion rates, and estimated blood loss in cerebral palsy patients undergoing proximal femoral varus derotational osteotomies.

Characteristic	No-TXA (n = 310)	TXA (n = 80)	*P* value
Preoperative Hb, g/dL	13.5 (1.2)	13.3 (1.3)	.27
Preoperative Hct	39.8 (3.1)	39.6 (3.4)	.61
Initial postoperative Hb, g/dL	9.5 (1.7)	9.5 (1.7)	.87
Initial postoperative Hct	28.0 (4.7)	28.3 (5.0)	.69
Change pre- to postoperative Hb, g/dL	−3.9 (2.0)	−3.8 (1.8)	.85
Change pre- to postoperative Hct	−11.8 (4.7)	−11.4 (5.1)	.58
Overall transfusion rate	78/310 (25.2%)	11/80 (13.8%)	.04^∗^
Intra-operative Transfusion rate	32/310 (10.3%)	6/80 (7.5%)	.53
Post-operative Transfusion rate	57/310 (18.4%)	6/80 (7.5%)	.02^∗^
Estimated blood loss, cc^∗∗^	177.4 (169.1)	142.9 (113.1)	.09
Percentage blood loss based on TBW, %^∗∗^	10.1 (8.7)	9.2 (7.0)	.40
Estimated blood Loss, cc^∗∗∗^	792.8 (515.5)	630.1 (500.7)	.07
Length of stay, days	2.9 (3.2)	2.9 (3.0)	.84

Values are presented as mean (standard deviation) unless otherwise noted; *P* values were determined using unpaired *t* tests and Fishers exact test for continuous and categorical variables, respectively.

∗ indicates significance at the *P* < .05 level.

∗∗ Estimated blood loss based on anesthesia records.

∗∗∗ Estimated blood loss using Hemoglobin balance method. Hb = hemoglobin, Hct = hematocrit, TBW = total body weight.

**Figure 1:**
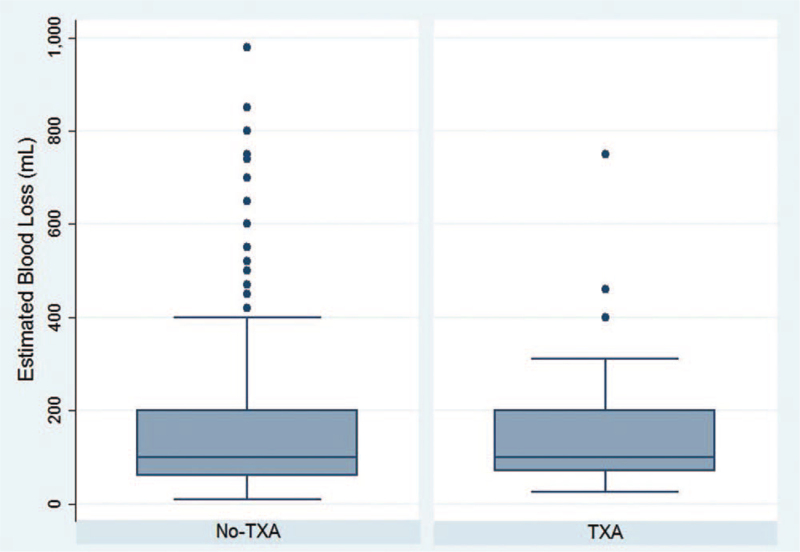
Intraoperative estimated blood loss (mL) in children with cerebral palsy undergoing proximal femoral varus derotational osteotomy (VDRO) surgery with and without tranexamic acid (TXA). Reproduced with permission from the Children's Orthopaedic Center, Los Angeles.

**Figure 2:**
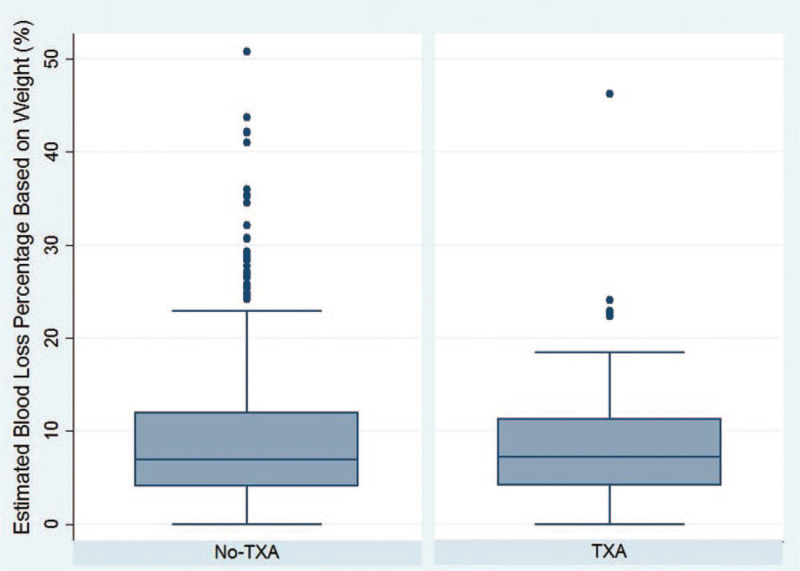
Intraoperative estimated blood loss percentage based on weight in children with cerebral palsy undergoing proximal femoral varus derotational osteotomy (VDRO) surgery with and without tranexamic acid (TXA). Reproduced with permission from the Children's Orthopaedic Center, Los Angeles.

The overall transfusion rate over the peri- and postoperative period was significantly lower in the TXA group (13.8%; 11/80) than in the No-TXA group (25.2%; 78/310) (*P* = .04; Table 2). Patients administered TXA were 0.55 times as likely to undergo transfusion over the peri- and postoperative period compared to patients who did not receive TXA (RR: 0.55; 95% CI: 0.31–0.98; *P* = .03). Therefore, the number needed to treat (NNT) with TXA to prevent a perioperative or postoperative transfusion in this series is 9. When controlling for unilateral vs bilateral VDROs, pelvic osteotomies, and ambulatory status, TXA administration was associated with a decreased overall transfusion rate (odds ratio (OR): 0.45; 95% CI: 0.21–0.96; *P* = .04). The intra-operative transfusion rates were similar between the 2 groups (TXA: 7.5% vs No-TXA: 10.3%; *P* = .53; Table 2). Patients were not less likely to undergo an intraoperative transfusion with TXA administration (RR: 0.73; 95% CI: 0.31–1.7; *P* = .45). The postoperative transfusion rate was significantly lower in the TXA group (7.5%; 6/80) than in the No-TXA group (18.4%; 57/310) (*P* = .02). Patients administered TXA were 0.41 times as likely to receive a postoperative transfusion than patients who were not administered TXA (RR: 0.41; 95% CI: 0.18–0.91; *P* = .02). Therefore, the NNT with TXA to prevent 1 postoperative transfusion in this series is 10. When controlling for unilateral versus bilateral VDROs, pelvic osteotomies, and ambulatory status, TXA administration was associated with a decreased postoperative transfusion rate (OR: 0.35; 95% CI: 0.14–0.89; *P* = .03). There was no statistical difference in EBL as recorded in the anesthesia records (TXA: 142.9 ± 113.1 mL versus No-TXA: 177.4 ± 169.1 mL; *P* = .09) (Fig. 1). Patients with both preoperative and immediate postoperative hemoglobin measurements had a significantly higher EBL as recorded in the anesthesia records (209.3 ± 174.6 mL) than patients without postoperative hemoglobin measurements (126.9 ± 128.3 mL) (*P* < .001). There was no difference in EBL between the TXA and No-TXA groups as determined via the hemoglobin balance method (TXA: 630.1 ± 500.7 mL vs No-TXA: 792.8 ± 515.5 mL; *P* = .07; Table 2). There was no difference in percentage of blood loss based on total body weight (TXA: 9.2 ± 7.0% vs No-TXA: 10.1 ± 8.7%; *P* = .40; Table 2) (Fig. 2). Postoperative hemoglobin and hematocrit levels were similar between the 2 groups (*P* = .87 and *P* = .69, respectively; Table 2). The change in preoperative to postoperative hemoglobin was not significantly different between those receiving TXA (-3.8 ± 1.8 g/dL) and those not receiving TXA (−3.9 ± 2.0 g/dL) (*P* = .85). The change in preoperative to postoperative hematocrit was not significantly different between those receiving TXA (−11.4 ± 5.1) and those not receiving TXA (−11.8 ± 4.7) (*P* = .58). Length of inpatient hospital stay was similar between the 2 groups (TXA: 2.9 ± 3.0 days vs No-TXA: 2.9 ± 3.2 days; *P* = .83). No major adverse events were associated with TXA use throughout the follow-up period (e.g. stroke, deep vein thrombosis, pulmonary embolism, etc.). Average length of follow-up for the TXA group was 13.1 ± 18.1 months while average length of follow-up for the No-TXA group was 37.7 ± 41.1 months (*P* < .001).

The first VDRO in a patient with CP during which TXA was administered occurred in January 2010. Following this, TXA was administered to 33% (80/244) of patients with CP undergoing VDRO. The intraoperative, postoperative, and overall transfusion rates for the 164 patients in the No -TXA group from 2010 to 2019 were comparable to the rates for the No-TXA group over the entire study period (Tables 3 and 4). In the 2010 to 2019 cohort, there was a significant decrease in postoperative transfusion rate (*P* = .03) and a trend toward decreased overall transfusion rate (*P* = .09) in the TXA group compared to the No-TXA group (Tables 3 and 4).

**Table 3:** Baseline information of cerebral palsy patients undergoing proximal femoral varus derotational osteotomies treated with and without tranexamic acid, in patients since TXA first began being administered.

Characteristic	No-TXA (n = 164)	TXA (n = 80)	*P* value
Gender, n (%)			
Male	106 (65)	38 (48)	
Female	58 (35)	42 (52)	.01^∗^
Weight at surgery, kg	28.7 (15.8)	24.6 (13.3)	.04^∗^
Height at surgery, cm	122.7 (24.3)	113.8 (24.1)	.01^∗^
GMFCS	I: 3	I: 1	
	II: 27	II: 9	
	III: 27	III: 10	.67
	IV: 56	IV: 33	
	V: 51	V: 27	
ASA Physical status	I: 3	I: 0	
	II: 43	II: 21	
	III: 118	III: 58	.40
	IV: 0	IV: 1	
Preoperative feeding status	Oral: 116	Oral: 51	
	G-tube: 37	G-tube: 26	
	GJ-tube: 4	GJ-tube: 2	.29
	Combined Oral/G-tube: 7	Combined Oral/G-tube: 1	
Preoperative seizure medication use	Yes: 68	Yes: 50	
	No: 96	No: 30	.003^∗^
VDRO side	Bilateral: 111	Bilateral: 67	.009^∗^
	Unilateral: 53	Unilateral: 13	
Pelvic osteotomy	Yes: 24	Yes: 10	
	No: 140	No: 70	.70
Procedures performed	6.2 (2.8)	5.6 (2.2)	.14
Operative time, minutes	234.5 (131.9)	234.5 (121.4)	.99

Values are presented as mean (standard deviation) unless otherwise noted; *P* values were determined using unpaired *t* tests and Fisher exact test for continuous and categorical variables, respectively.

∗ indicates significance at the *P* < .05 level. ASA = American Society of Anesthesiologists, G-tube = gastrostomy tube, GJ-tube = gastro-jejunal tube, GMFCS = Gross Motor Function Classification System, VDRO = varus derotational osteotomy.

**Table 4:** Pre- and postoperative laboratory values, transfusion rates, and estimated blood loss in cerebral palsy patients undergoing proximal femoral varus derotational osteotomies, in patients since TXA first began being administered.

Characteristic	No-TXA (n = 164)	TXA (n = 80)	*P* value
Preoperative Hb, g/dL	13.4 (1.2)	13.3 (1.3)	.63
Pre-operative Hct	39.7 (3.1)	39.6 (3.4)	.79
Initial postoperative Hb, g/dL	9.5 (1.6)	9.5 (1.7)	.97
Initial postoperative Hct	28.1 (4.7)	28.3 (5.0)	.85
Change pre- to postoperative Hb, g/dL	−3.9 (1.7)	−3.8 (1.8)	.89
Change pre- to postoperative Hct	−11.6 (4.8)	−11.4 (5.1)	.76
Overall transfusion rate	39/164 (23.8%)	11/80 (13.8%)	.09
Intra-operative Transfusion rate	16/164 (9.8%)	6/80 (7.5%)	.64
Postoperative Transfusion rate	29/164 (17.7%)	6/80 (7.5%)	.03^∗^
Estimated blood loss, cc^∗∗^	168.7 (168.7)	142.9 (113.1)	.22
Percentage blood loss based on TBW, %^∗∗^	9.3 (9.1)	9.2 (7.0)	.88
Estimated blood loss, cc^∗∗∗^	795.2 (541.0)	630.1 (500.7)	.09
Length of stay, days	2.7 (2.8)	2.9 (3.0)	.75

Values are presented as mean (standard deviation) unless otherwise noted; *P* values were determined using unpaired *t* tests and Fisher exact test for continuous and categorical variables, respectively.

∗ indicates significance at the *P* < .05 level.

∗∗ Estimated blood loss based on anesthesia records.

∗∗∗ Estimated blood loss using Hemoglobin balance method. Hb = hemoglobin, Hct = hematocrit, TBW = total body weight.

## 4 Discussion

With 80 patients who received TXA intraoperatively, this study is the largest involving TXA administration in patients with CP. In the current series, the overall transfusion rate (for the entire hospitalization) of 13.8% in those who received TXA was significantly less than the rate of 25.2% in those who did not, and there was also a significantly lower rate of postoperative transfusion in the TXA group (7.5%) than in the No-TXA group (18.4%). The NNT with TXA to avoid one blood transfusion for the entire hospitalization was 9, and to avoid a postoperative transfusion was 10. There were no thromboembolic complications in these 80 patients at a mean follow-up of over 1 year, indicating that TXA administration appears safe and effective in this patient population.

There are relatively few additional studies examining intra-operative TXA use in patients with CP. In a prior study at our institution by Nazareth et al, the authors examined patients with CP undergoing VDRO.^[14]^ In that study, patients treated with TXA tended to have lower blood loss and transfusion rates, but the findings were not statistically significant because the study was underpowered. The present investigation extended the time frame in order to increase the sample size and the power of the prior study (390 total patients vs 258, including 80 vs 36 treated with TXA), in an attempt to elucidate whether TXA administration truly altered transfusion rates.

In a recent of study of 119 patients undergoing hip reconstruction (47 of whom received TXA), Lins et al similarly reported decreased post-operative transfusion rates in the TXA group, but comparable intraoperative transfusions rates in the 2 groups.^[34]^ These findings are similar to those in the current study, though Lins et al did not report overall transfusion rate, and they reported post-operative transfusion rates much higher than those in the current study, including 32% in the patients who had received TXA and 47% in those who had not.^[34]^

In a study of 84 patients with CP undergoing posterior spinal fusion with instrumentation, Dhawale et al determined that anti-fibrinolytic agents, including TXA, effectively reduced blood loss and cell salvage transfusion.^[35]^ Sethna et al studied 44 pediatric patients undergoing spinal fusion, 23 of whom received TXA, and found a 41% decrease in EBL (1,230 mL vs 2,085 mL) for those receiving TXA compared to placebo.^[35,36]^ In contrast to these studies, Majid et al studied 51 hip reconstructions in children with CP, and did not find significant differences between patients who did and did not receive TXA, but reported a trend toward higher transfusion rate, lower postoperative hemoglobin, and longer hospital stay in the TXA group.^[12]^ Their results were confounded by the fact that the patients who received TXA had more severe disease as they had lower GMFCS levels and were more likely to undergo bilateral hip reconstructions with concomitant pelvic and femoral osteotomies.^[12]^

In the current study, though the intraoperative EBL was similar in the TXA and No-TXA group, the No-TXA group had a much larger standard deviation indicating more variability in the EBL in these patients, and there were a large number of high EBL outliers in the No-TXA group. The No-TXA group had 10 patients (3.2%) experience blood loss of greater than 600 mL, while the TXA group had only 1 patient (1.3%) experience at least 600 mL of blood loss. Our findings parallel those of Nazareth et al from our institution, in a smaller cohort.^[14]^ The average blood loss in our No-TXA patients in absolute terms and in blood loss per kilogram was lower than those in studies from other institutions,^[14,15]^ but we were still able to significantly reduce blood transfusion by the use of TXA in our study patients. Previous studies involving spinal surgery in children with CP have also found that EBL is significantly reduced with TXA administration, though the EBL in those studies was markedly higher (1230–2685 mL) than in our study.^[35,36]^

The validity and reliability of EBL measurements, which are typically estimated based upon suction volume, sponge counts and surgeon estimations, have been questioned.^[37,38]^ As a result, we decided to also evaluate EBL using the hemoglobin balance method,^[30–33]^ which resulted in EBL values which were substantially higher than those estimated from anesthesia records (Table 2). Part of the increased EBL using the hemoglobin balance method appears to be due to the lack of uniformity in pre- and postoperative hemoglobin monitoring, with both pre- and postoperative hemoglobin measurements available in only 53.8% (43/80) of patients in the TXA group and 52.6% (163/310) patients in the No-TXA group. At our institution, patients with higher EBL have hemoglobin checks postoperatively, while those with low EBL (EBL <5%–8% of blood volume) do not; this is borne out by the fact that the mean EBL estimated in the anesthesia record in patients who had postoperative blood draws was 65% greater (209.3 mL vs 126.9 mL) than did those who did not. As such, the hemoglobin balance method was only used in the patients whose EBL in the medical record was estimated at 65% greater than in patients in whom is was not used. As the proportion of patients in the TXA group and No-TXA group who had post-operative hemoglobin measurements is similar, it is unlikely that surgeons in the TXA group were biased to pay attention to hemostasis due to patient factors, medical comorbidities, or surgical procedure (unilateral vs bilateral VDRO, pelvic osteotomy, etc.). Although it did not reach significance, the TXA group tended to have a lower estimated blood loss than the No-TXA group both using the hemoglobin balance method and using the EBL recorded in the medical record.

The overall transfusion rate during the intra- and postoperative period in the no-TXA group was 25.2%, which is considerably lower than the transfusion rates of 37% to 67% typically reported in the literature.^[12,14,15]^ Despite the low transfusion rates in the No-TXA group, the risk of transfusion was significantly lower in the TXA group. Although an a priori power analysis was not performed, the 95% CI for overall transfusion rate and post-operative transfusion rate indicate that the risk of transfusion is likely lowered by TXA administration, despite the disproportionate sample sizes between the 2 groups. Additionally, when controlling for unilateral vs bilateral VDROs, pelvic osteotomies, and ambulatory status, TXA administration was still significantly associated with a decreased overall and post-operative transfusion rate, indicating that possible heterogeneity of surgical and patient factors between the 2 groups was not responsible for the decreased transfusion rate. Though there was a trend towards lower intra-operative transfusion rate, this was not significant, likely do the rarity of intra-operative transfusion in both groups coupled with the small absolute risk reduction of intra-operative transfusion with TXA administration (2.8%). Thus, given our sample sizes, we were likely underpowered to detect a difference in intra-operative transfusion rates. The small absolute risk reduction elucidated may not be a true difference and warrants further investigation. Throughout the entire study period, surgical and anesthesia technique, surgeon experience, and medical optimization might have had an impact on blood loss and transfusion rates.

In order to assess whether the lower transfusion rates in the TXA group were due to improvements in surgical and anesthesia technique over time, we compared the data from patients operated on before and after the advent of TXA use at our institution in 2010. Of the final 244 patients, 80 (33%) received TXA and 164 (67%) did not. The data from these final 164 patients in the No-TXA were compared to the data from the entire cohort of 310 patients in the No-TXA group, and the rates of transfusion intra-operatively, postoperatively, and for the entire hospital stay are comparable. Further, when evaluating just the cohort from 2010 onward, the TXA group had lower rates of both postoperative transfusion and overall transfusion than did those in the No-TXA group, though only the postoperative transfusion rate (*P* = .03) was statistically significant due to the smaller sample size. These data all point to the important impact of TXA on blood loss and transfusion rates in children with CP undergoing hip reconstruction.

Importantly, the patients treated with TXA were more likely to be using preoperative seizure medications (63% TXA group and 41% No-TXA), and had higher rates of feeding tube dependence (36% TXA group and 26% No-TXA). Seizure medication use and poor nutritional status have both been identified as risk factors for increased blood loss during surgery in patients with CP.^[9,10]^ Additionally, patients in the TXA group were more likely to undergo bilateral VDROs, indicating more extensive surgery, with the potential for increased blood loss and transfusion requirements. The increased disease severity in the TXA group, along with the increased rate of bilateral surgery, may have minimized the observed therapeutic effect of TXA on reducing blood loss and transfusion requirements. Despite this, there was still a significant reduction in transfusion rate and a trend towards decreased EBL in the TXA cohort.

The optimal dose and timing of TXA administration to achieve therapeutic effects, while minimizing the potential for adverse events remains unclear. The average dose of intraoperative TXA was 54.0 mg/kg in our study, however the loading and maintenance dose protocol changed throughout the study period. This is similar to the 40.8 mg/kg reported by Dhawale et al in patients with CP undergoing posterior spinal fusions for scoliosis,^[35]^ and similar to that reported by Lins et al *l* who reported a loading dose of 10 to 30 mg/kg followed by a continuous infusion of 5 to 10 mg/kg/hour thereafter during surgery.^[34]^ In a study of 36 pediatric hospitals across the United States, Nishijima et al noted that the average dose of TXA was 22.4 mg/kg in all pediatric patients undergoing congenital heart surgery, scoliosis, or craniofacial surgery.^[39]^ The retrospective nature of this study and the variability in intraoperative dosing limits our ability to determine the optimal dosing and timing of TXA administration intraoperatively. This warrants further investigation in a high-powered, randomized controlled trial.

The study is limited by its retrospective nature. While transfusion rates, hemoglobin, and hematocrit levels are accurately recorded at our institution, there was no protocol for pre- and postoperative laboratory monitoring. Postoperative hematocrit and hemoglobin measurements may have been confounded by induced hypotension and fluid administration intra-operatively resulting in hemodilution. However, the anesthesia team used these techniques routinely; therefore, they were likely applied to the cohorts in a similar manner and are unlikely to change the results of the study. Also, there was no protocol for transfusion and physicians may have had different thresholds for transfusing patients. Additionally, wound drains are not routinely placed during VDRO surgery at our institution, so postoperative blood loss cannot be accurately quantified.

In conclusion, the use of intra-operative TXA was associated with a significant decrease in the overall transfusion rate and post-operative transfusion rate in patients with CP undergoing VDRO. Though there was a trend towards lower intraoperative transfusion rate, this was not significant, likely do the infrequency of intraoperative transfusion in both groups. The present study is the largest to date of patients with CP treated with TXA while undergoing an orthopedic procedure. Based on our results, administration of TXA intraoperatively for patients with CP undergoing VDRO surgery appears to be safe and can effectively reduce blood loss and transfusion requirements.

## Author contributions

**Conceptualization:** Rachel Y. Goldstein, Lydia Andras, Robert M. Kay.

**Data curation:** Edward Compton, Alexander Nazareth, Stephen J. Shymon.

**Formal analysis:** Edward Compton.

**Investigation:** Rachel Y. Goldstein, Robert M. Kay.

**Methodology:** Rachel Y. Goldstein, Lydia Andras, Robert M. Kay.

**Supervision:** Rachel Y. Goldstein, Robert M. Kay.

**Visualization:** Robert M. Kay.

**Writing – original draft:** Edward Compton.

**Writing – review & editing:** Edward Compton, Rachel Y. Goldstein, Alexander Nazareth, Stephen J. Shymon, Lydia Andras, Robert M. Kay.
